# Test Ordering and Completion During Virtual vs In-Person Annual Visits

**DOI:** 10.1001/jamanetworkopen.2026.0013

**Published:** 2026-02-25

**Authors:** Ishani Ganguli, Nicholas E. Daley, Ateev Mehrotra, Meredith B. Rosenthal, David M. Cutler

**Affiliations:** 1 Harvard University, Boston, Massachusetts; 2 Division of General Internal Medicine and Primary Care, Brigham and Women’s Hospital, Boston, Massachusetts; 3 Department of Health Services, Policy and Practice, Brown University School of Public Health, Providence, Rhode Island; 4 National Bureau of Economic Research, Cambridge, Massachusetts

## Abstract

**Importance:**

Telemedicine use has been associated with lower rates of overall testing and low-value testing, but the underlying mechanisms are unknown.

**Objective:**

To compare medical screening test ordering and completion rates between in-person and virtual annual visits, and to determine how these rates varied by test value (high vs low) and test type (point of care vs scheduled).

**Design, Setting, and Participants:**

This cohort study used electronic health record data on virtual and in-person annual visits conducted between January 1, 2022, and October 25, 2023, among adult patients at 87 primary care practices in a large health system. Data were analyzed from September 2024 to June 2025.

**Exposure:**

Virtual or in-person annual visit.

**Main Outcomes and Measures:**

Rates of ordering and completion of 15 high- and low-value tests within 11 months after visit.

**Results:**

A total of 22 547 matched in-person and virtual visits were examined involving 20 948 patients (mean [SD] age, 51.0 [15.9] years, 14 502 women [69.2%]). All 15 high- and low-value tests were more likely to be ordered and completed at in-person vs virtual visits; generally, high-value tests were more likely to be ordered and completed than low-value tests. At virtual compared with in-person visits, high-value tests were 14.3% (95% CI, −15.5% to −13.2%) less likely to be ordered (relative to the 54.8% in-person rate) and 13.1% (95% CI, −14.1% to −12.0%) less likely to be completed (relative to the 87.7% in-person rate). Low-value tests were 19.3% (95% CI, −21.0% to −17.5%) less likely to be ordered (27.8% in-person rate) and 17.3% (95% CI, −18.5% to −16.1%) less likely to be completed (91.1% in-person rate). When compared with scheduled tests, point-of-care laboratory tests had larger decreases at virtual versus in-person visits in ordering (−18.5% [95% CI, −19.9% to −17.1%] relative to 37.7% in person, vs −11.6% [95% CI, −13.6 to −9.6] for scheduled tests [26.8% in person]) and completion (−16.3% [95% CI, −17.3 to −15.3]; 93.7% in person, vs −6.2% [95% CI, −8.9 to −3.4]; 63.0% in person).

**Conclusions and Relevance:**

In this cohort study, high- and low-value tests were less likely to be both ordered and completed at virtual annual visits compared with in-person annual visits, particularly low-value tests and laboratory tests. Telemedicine may introduce frictions for clinicians and patients that differentially reduce low-value testing, although high-value tests were also affected; health systems could consider tools, such as gap closure alerts, to selectively promote high-value care.

## Key Points

**Question:**

Among virtual vs in-person annual visits, how do rates of test ordering and test completion differ overall and by test value and type?

**Findings:**

In this cohort study of 22 547 annual visits, high- and low-value tests were less likely to be both ordered and completed at virtual compared with in-person visits, with slightly larger decreases for low-value (compared with high-value) tests and for point-of care laboratory (compared with scheduled) tests.

**Meaning:**

The findings suggest that telemedicine may introduce frictions for clinicians and patients that differentially reduce low-value testing, although high-value tests are also affected, clarifying both a potential benefit for the virtual modality and the need for targeted tools to selectively promote high-value testing.

## Teaser

This cohort study compares the rates of test ordering by clinicians and test completion by patients at virtual vs in-person annual primary care visits.

## Introduction

Telemedicine (ie, synchronous audio and video visits) remains a widely used visit modality in post–COVID-19 pandemic years.^1,2^ Yet there is active policy debate about continuing telemedicine coverage by Medicare and other payers, hinging in part on how telemedicine affects care quality and spending. While the virtual modality precludes many aspects of the physical examination, virtual visits offer patients convenience and accessibility and may lower overall visit spending.^3,4,5,6,7,8,9^ Telemedicine may also have spillover effects on medical testing. For example, this approach might theoretically help to reduce the persistent and costly problem of low-value care, defined as medical tests and other services that offer minimal benefit yet have potential for direct and cascading harms.^10,11,12,13,14^

Prior studies have found that telemedicine use may be associated with lower rates of overall testing^5,15,16,17,18,19^ and of low-value testing in particular,^3,20^ but the underlying mechanisms are unclear. For instance, Medicare claims–based studies found that patients receiving care in Michigan primary care practices^20^ and in US health systems^3^ with greater vs lesser telemedicine adoption were less likely to receive low-value tests, such as cervical cancer screenings in older women and screening metabolic panels. In theory, this finding might be due to the virtual modality creating barriers to completing tests; for example, if patients are not in the office, clinicians may be less likely to order tests or patients may be less likely to complete them.^5,16,17,18,19,21^ The differences between virtual and in-person visits might be greater for point-of-care tests (performed in the examination room or at an on-site laboratory) than for scheduled tests. Similarly, the differences might be greater for low-value than for high-value tests, as clinicians or patients may judge low-value tests to be less worthy of any extra logistical effort conferred by the virtual modality. Supporting this hypothesis is a Medicare claims–based study that found patients in US health systems with greater vs lesser telemedicine adoption received high-value (ie, guideline-recommended) breast, colorectal, prostate, and cervical cancer screening tests at equivalent rates.^4^ However, prior studies either have not examined testing by value or type or have lacked the granularity to observe tests that were ordered but not completed or to distinguish between the roles of clinicians and patients in test use.

Understanding the mechanisms of how telemedicine may affect testing behaviors would inform policy decisions and the implementation of telemedicine in clinics and health systems. In this study, we used electronic health record (EHR) data from a large health system to compare medical screening test ordering and completion rates between in-person and virtual annual visits, and to determine how these rates varied by test value (high vs low) and test type (point of care vs scheduled). We focused on annual visits because there are a range of point-of-care and scheduled tests that can be, and often are, provided in these visits that are well-defined in prior literature as high value or low value.^14,22,23,24^

## Methods

### Data Source

The Mass General Brigham (MGB) is a large health system in New England. We extracted data from the MGB Enterprise Data Warehouse, which includes detailed structured and unstructured information on in-person visits and virtual encounters, test orders and results, patient demographics, patient zip codes, and diagnostic and billing codes from the EHR. We performed iterative medical record reviews to confirm the validity of the data extract elements and outcome measures (eMethods in Supplement 1). The MGB Institutional Review Board deemed this study exempt from ethics review and informed consent requirement in accordance with 45 CFR § 46.116(f). We followed the Strengthening the Reporting of Observational Studies in Epidemiology (STROBE) reporting guideline.

### Study Cohort

We identified all annual visits (defined as visits with an annual visit text label [eMethods in Supplement 1] and one of the following billing codes: G0438, G0439, 99385-99387, 99395-99397, or 99213-99215) that were completed across 234 MGB primary care practices between January 1, 2022, and October 25, 2023 (the last date for which we had 11 months of follow-up data), for adult patients (aged ≥18 years as of visit date) with an MGB primary care physician (PCP, defined by the EHR PCP field as of that visit). We excluded all patient visits for patients with more than 2 annual visits in 2022 through 2023 or with 2 annual visits within 180 days since these patients may experience atypical testing patterns. We then identified annual visits as virtual or in-person based on EHR text fields corroborated by medical record review (eMethods in Supplement 1).

### Measures

#### Outcomes

The outcomes were rates of orders and completions (within 11 months after order date, to avoid overlap with the subsequent annual visit) of 13 tests commonly ordered during annual visits (eTable 1 in Supplement 1). We defined a total of 15 tests as high value or low value in specific clinical scenarios based on guidelines (eg, ABIM Foundation’s Choosing Wisely campaign^25,26^ and US Preventive Services Task Force^27^); cervical cancer and colorectal cancer (CRC) screening were each represented twice, as they could be defined as high value or low value depending on the clinical scenario. We operationalized these definitions using EHR-based demographic information (age and sex); *International Statistical Classification of Diseases and Related Health Problems, Tenth Revision* diagnosis codes billed to visits or on problem lists; and health maintenance modifiers (clinicians enter these into their patients’ medical records to capture clinical nuances that are then reflected in EHR alerts—eg, Papanicolaou test due every 5 years).

High-value tests included cervical cancer screening (for women aged 21-65 years), Hemoglobin A1c (HbA_1c_), lipid testing, CRC screening (for patients aged 45-75 years), and mammography (for women aged 40-74 years). Low-value tests included cervical cancer screening (for women aged >65 years),^28^ screening electrocardiogram (ECG),^14,28^ screening basic metabolic panel, screening liver function test, screening comprehensive metabolic panel, screening complete blood count with or without differential, prostate cancer screening (prostate-specific antigen test; for men aged >70 years),^28,29^ thyroid screening (thyroid-stimulating hormone), screening urinalysis,^14^ and CRC screening (for patients aged >85 years). We used these designations to identify tests as high or low value. We also used them to identify visits as eligible for a given high- or low-value test to mitigate confounding by indication for a given visit modality (eg, if a patient were eligible for a high-value lipid screening, they or their clinician may be more likely to pick an in-person visit).

In addition, we identified tests by their type. Point-of-care tests included examination room (cervical cancer screening and screening ECG) and laboratory (HbA_1c_, lipid, metabolic panel, complete blood count, prostate cancer screening, thyroid screening, and urinalysis) tests. Scheduled tests included CRC screening and mammography.

For a given visit, we ascertained whether each of these tests was ordered—defined as a test order that was either linked to the index visit encounter or placed by the clinician who provided the index visit (identified by National Provider Identifier) within a 9-day period (7 days before to 1 day after the visit, as in prior work^14^)—and noted the order date. We then determined whether the test was completed (ie, result recorded in the EHR) in the subsequent 11 months and, if so, noted the completion date.

The secondary outcome was time to test completion, calculated as test completion date minus test order date. See eMethods and eTable 2 in Supplement 1 for details.

#### Patient and Visit Characteristics

We captured a series of patient and visit characteristics that were potential confounders of the relationship between visit modality and test ordering or completion (eg, distance from clinic [since patients living farther from the clinic may be more likely to have a telemedicine visit and less likely to complete tests] and patient portal engagement) or were linked to testing adherence (eg, insurance status) such that their inclusion in models would improve precision.

Patient characteristics were age, sex, self-reported primary patient language, self-reported race, self-reported ethnicity, insurance type, Charlson Comorbidity Index (measured using all active diagnoses on the patient’s EHR problem list), distance from residence to clinic (estimated using zip codes of patient residence and clinical site), patient portal engagement (defined as at least 1 portal login within a year before the visit), previous annual visit (within 24 months prior to index visit), and previous virtual annual visit (within 24 months prior to index visit). Race and ethnicity data were collected in this study because studies have linked race and ethnicity with telemedicine use and preventive service use. Racial and ethnic categories analyzed were Asian, Black, Hispanic, White, and other (including American Indian or Alaska Native, Native Hawaiian or Other Pacific Islander, and other races not listed in the EHR).

Visit characteristics were the providing clinician, whether the visit was with the patient’s PCP, whether the visit was co-billed as a problem-based visit (ie, presence of level 25 modifier), visit year, and quarter (eg, quarter 1: January to March).

### Statistical Analysis

#### Matching

We built a propensity score model to estimate the propensity for a visit to be virtual, using all patient and visit characteristics other than clinician and visit year. Then, for every virtual visit, we propensity score–matched the virtual visit to up to 5 in-person visits with the exact same clinician and visit year(to control for differences in practice style^30^ and temporal trends). Specifically, we used sequential 1:5 nearest neighbor propensity matching without replacement and then weighted the matched in-person visits by the inverse of the number of in-person visits matched to the given virtual visit (eg, for a virtual visit with 5 matches, each matched in-person visit received a weight of 1/5). We calculated standardized mean differences (SMDs) to assess balance between the virtual visit and in-person visit groups before and after matching. Characteristics that did not achieve sufficient balance (defined as SMD <0.10) were included as covariates in subsequent models.

#### Analyses

We performed patient-visit level analyses. We described the distribution of tests ordered per visit. For each test, we used log-binomial regression to compare test ordering between virtual and in-person visits (among all visits) and Poisson regression with robust error variance to compare test completion between virtual and in-person visits (among all visits in which the test was ordered); we used Poisson regression because log-binomial models often failed to converge for these high-probability outcomes,^31^ We used survival analysis to compare time to test completion between virtual and in-person patient visits (represented as hazard ratios [HRs]).

We applied the clinical criteria (eg, sex, age, and relevant diagnoses) to identify tests as either high or low value and to identify visits eligible for each high- and low-value test. Next, among eligible visits, we compared virtual with in-person visits on rates of test ordering, test completion, and time to completion. We examined adjusted differences in these outcomes between virtual and in-person visits. When needed, we adjusted weights to account for fewer matched in-person visits. To compare groups of tests by their value (high vs low) and separately, by their type (point of care, which we limited to laboratory tests in these analyses given the infrequency of examination room tests ordered at virtual visits, vs scheduled), we used binomial models to compare ordering rates and Poisson regression (offset by the natural log of orders) to compare completion rates contingent on ordering (primary models). The eMethods in Supplement 1 provides details.

Models were adjusted for the covariates, and SEs were clustered by patient. We performed analyses using R, version 4.4.2 (R Project for Statistical Computing). Two-sided *P* < .05 represented statistical significance. We used Holm-Bonferroni to correct the primary analyses for multiple comparisons and presented CIs throughout to allow interpretation of clinical significance. Data were analyzed from September 2024 to June 2025.

## Results

Our final sample before matching included 439 581 visits with 329 687 patients across 234 clinics; 4596 (1.0%) of these visits were virtual. After matching, the sample included 22 547 visits from 20 948 patients (mean [SD] age, 51.0 [15.9] years; 14 502 women [69.2%] and 6446 men [30.8%]) across 87 clinics; 3887 (17.2%) of these visits were virtual (eFigure in Supplement 1).

Patient and visit characteristics in the virtual and in-person visit groups were similar before and after matching, with SMD less than 0.10 for all characteristics other than insurance type, prior virtual annual visit, and visit year quarter (Table 1). Across matched in-person and virtual visit groups, 96.7% (18 089) to 97.6% (3795) of patients spoke English as their primary language, and race and ethnicity were self-reported as follows: 1698 (9.1%) and 309 (8.0%) Asian, 981 (5.3%) and 182 (4.7%) Black, 1132 (6.2%) and 212 (5.5%) Hispanic, 14 610 (78.0%) and 3134 (80.6%) White, and 923 (5.1%) and 167 (4.3%) other individuals, respectively. Most visits (2917 [75.0%] and 14 746 [79.3%]) were covered by commercial insurance, and most visits (16 213 [87.2%]) were with the patients’ own PCPs.

**Table 1.** Patient and Visit Characteristics

Characteristic	All visits, No. (%)			Matched visits, No. (%)		
	Virtual (n = 4596)	In-person (n = 434 985)	SMD^a^	Virtual (n = 3887)	In-person (n = 18 660)	SMD^a^
Patient characteristics						
Age, mean (SD), y	51.3 (17.2)	51.3 (16.0)	0.00	51.7 (17.1)	50.7 (15.5)	0.06
Sex^b^						
Female	3228 (70.2)	270 378 (62.2)	0.17	2752 (70.8)	12 976 (69.3)	0.03
Male	1368 (29.8)	164 590 (37.8)	0.17	1135 (29.2)	5684 (30.7)	0.03
Primary patient language						
English	4475 (97.4)	417 846 (96.1)	0.07	3795 (97.6)	18 089 (96.7)	0.05
Spanish	43 (0.9)	8732 (2.0)	0.08	29 (0.8)	198 (1.2)	0.04
Other	66 (1.4)	6110 (1.4)	0.00	57 (1.5)	338 (1.9)	0.03
Race^c^						
Asian	353 (7.7)	26 574 (6.1)	0.04	309 (8.0)	1698 (9.1)	0.04
Black	242 (5.3)	21 454 (4.9)	0.02	182 (4.7)	981 (5.3)	0.03
White	3669 (79.8)	354 411 (81.5)	0.04	3134 (80.6)	14 610 (78.0)	0.06
Other^d^	217 (4.7)	20 960 (4.8)	0.00	167 (4.3)	923 (5.1)	0.04
Ethnicity^c^						
Hispanic	282 (6.1)	32 210 (7.4)	0.05	212 (5.5)	1132 (6.2)	0.03
Insurance type						
Commercial	3445 (75.0)	341 115 (78.4)	0.08	2917 (75.0)	14 746 (79.3)	0.10
Medicare	976 (21.2)	55 159 (12.7)	0.26	832 (21.4)	3116 (16.4)	0.13
Self-pay	128 (2.8)	30 876 (7.1)	0.17	98 (2.5)	527 (2.8)	0.02
Other^e^	47 (1.0)	6433 (1.5)	0.04	40 (1.0)	270 (1.5)	0.04
CCI, mean (SD)	2.0 (2.3)	1.9 (2.1)	0.07	2.0 (2.3)	1.8 (2.1)	0.08
Distance from residence to clinic, mean (SD), miles	36.0 (192.0)	22.3 (141.2)	0.10	38.0 (200.8)	37.4 (215.1)	0.00
Patient portal login in prior year	4557 (99.2)	409 214 (94.1)	0.22	3856 (99.2)	18 424 (98.6)	0.05
Any annual visit in prior 24 mo	2608 (56.7)	308 910 (71.0)	0.31	2419 (62.2)	11 613 (62.3)	0.00
Any virtual annual visit in prior 24 mos	916 (19.9)	8864 (2.0)	1.22	782 (20.1)	2719 (14.5)	0.16
Visit characteristics						
With patient’s PCP	3974 (86.5)	358 053 (82.3)	0.11	3399 (87.4)	16 213 (87.2)	0.01
Co-billed as a problem-based visit	620 (13.5)	103 718 (23.8)	0.24	609 (15.7)	3163 (17.7)	0.05
Visit quarter						
Quarter 1 (Jan-Mar)	1844 (40.1)	119 887 (27.6)	0.28	1542 (39.7)	6527 (34.4)	0.11
Quarter 2 (Apr-Jun)	1179 (25.7)	117 410 (27.0)	0.03	1015 (26.1)	5330 (28.6)	0.05
Quarter 3 (Jul-Sep)	932 (20.3)	115 991 (26.7)	0.14	791 (20.3)	4065 (21.7)	0.03
Quarter 4 (Oct-Dec)	641 (13.9)	81 697 (18.8)	0.12	539 (13.9)	2738 (15.3)	0.04

Abbreviations: CCI, Charlson Comorbidity Index; NA, PCP, primary care physician: SMD, standardized mean difference.

^a^ SMDs measure the balance in baseline covariates between 2 groups. Values less than 0.10 suggest that the groups are well-balanced.

^b^ Sex unknown for 17 patients (all in-person visits).

^c^ Race and ethnicity were self-identified and obtained from the electronic health record (EHR). Race was unknown for a total of 11 701 patients across all visits and 543 patients across matched visits. Ethnicity was unknown for 32 599 patients across all visits and 1856 patients across matched visits.

^d^ Other includes American Indian or Alaska Native, Native Hawaiian or Other Pacific Islander, and races not listed in the EHR.

^e^ Other includes Medicaid, other government insurance, free care, international, and Workers’ Comp or motor vehicle insurance. Insurance data missing for 1392 patients across all visits and 1 patient across matched visits.

**Figure 1.**
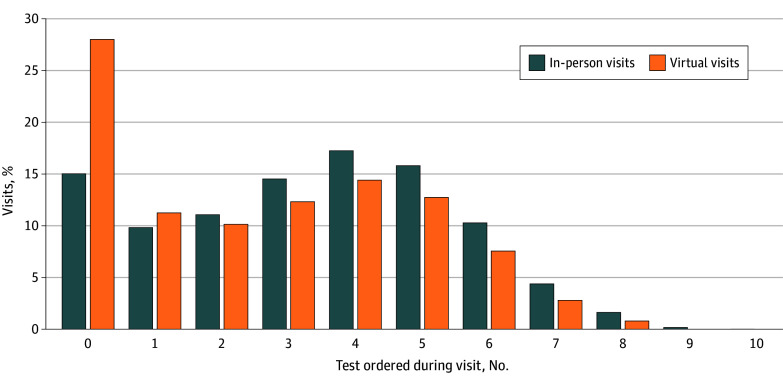
Histogram of Tests Ordered During In-Person and Virtual Annual Visits There were no visits in which more than 10 tests (of the 13 examined without value designations) were ordered.

Overall, the number of tests ordered per annual visit were higher for in-person than virtual visits (Figure 1). Clinicians ordered a mean (SD) number of 3.3 (2.2; median [IQR], 3 [2-5]) tests at in-person visits and 2.6 (2.2; median [IQR], 3 [0-4]) tests at virtual visits.

### Ordering and Completion by Test Value and Type

**Figure 2.**
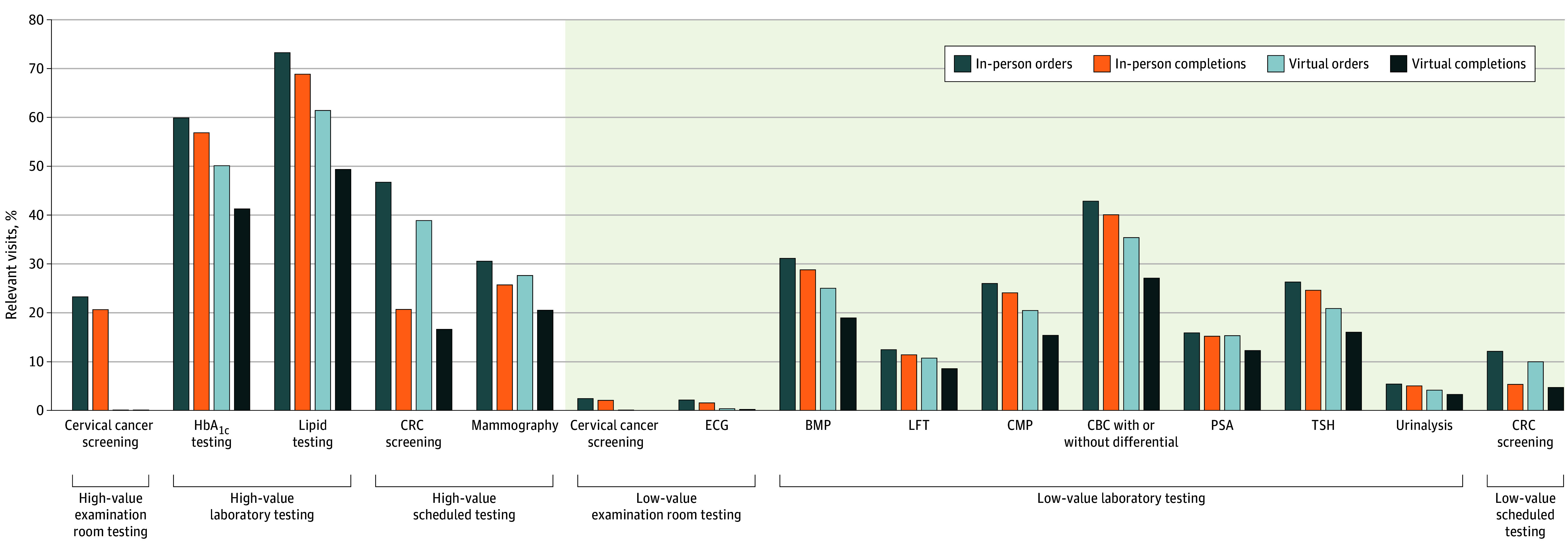
Graph of Order and Completion Rates of High-Value and Low-Value Screening Tests Among Relevant Visits Relevant visits are those eligible for a given test. See for clinical criteria in eMethods and eTable 2 in Supplement 1. BMP indicates basic metabolic panel; CBC, complete blood count; CMP, comprehensive metabolic panel; CRC, colorectal cancer; ECG, electrocardiogram; HbA_1c_, hemoglobin A_1c_; LFT, liver function test; PSA, prostate-specific antigen (for prostate cancer screening); TSH, thyrotropin (formerly thyroid-stimulating hormone).

When examining tests specified as high value or low value in the context of relevant visits (ie, those eligible for a given test), high-value tests were generally ordered and completed at higher rates than low-value tests (Figure 2; eTable 3 in Supplement 1). Across high- and low-value tests, ordering and completion rates were lower for virtual visits than for in-person visits (Figure 2). At virtual visits, tests normally conducted in the examination room (cervical cancer screenings and screening ECGs) were rarely ordered.

### Adjusted Analyses

For nearly all tests examined, tests were significantly less likely to be ordered at virtual vs in-person visits (Table 2). High-value tests were 14.3% (95% CI, −15.5% to −13.2%) less likely to be ordered and 13.1% (95% CI, −14.1% to −12.0%) less likely to be completed in virtual visits vs in-person visits (relative to a 54.8% in-person ordering rate and 87.7% in-person completion rate). Low-value tests were 19.3% (95% CI, −21.0% to −17.5%) less likely to be ordered and 17.3% (95% CI, −18.5% to −16.1%) less likely to be completed in virtual visits vs in-person visits (relative to a 27.8% in-person ordering rate and 91.1% in-person completion rate). Point-of-care laboratory tests had larger differences by visit modality for both ordering and completion than scheduled tests. Specifically, point-of-care laboratory tests were 18.5% (95% CI, −19.9% to −17.1%) less likely to be ordered and 16.3% (95% CI, −17.3% to −15.3%) less likely to be completed in virtual visits than in-person visits (relative to a 37.7% in-person ordering rate and 93.7% in-person completion rate). For scheduled tests, these decreases were 11.6% (95% CI, −13.6% to −9.6%) for ordering and 6.2% (95% CI, −8.9% to −3.4%) for completion (relative to a 26.8% in-person ordering rate and 63.0% in-person completion rate). These results varied across the scheduled tests examined. While high-value mammograms were less likely to be completed when ordered during virtual visits (−11.9%; 95% CI, −16.6% to −6.9%), high-value and low-value CRC screenings were not (−4.1% [95% CI, −16.1% to 9.6%] and 8.7% [95% CI, −9.4% to 30.3%]).

**Table 2.** Differences in Test Ordering and Completion Between In-Person and Virtual Visits

Test	% Of eligible visits with a test order, in-person visit	Adjusted relative % difference in test order rate (95% CI), virtual vs in-person visit	% Of visits with a test completion (contingent on order), in-person visit	Adjusted relative % difference in test completion rate (contingent on order) (95% CI), virtual vs in-person visit	Days to test completion, median (IQR)	
					In-person visit	Virtual visit
Composite test categories						
High value^a^	54.8	−14.3 (−15.5 to −13.2)^b^	87.7	−13.1 (−14.1 to −12.0)^b^	0 (0 to 8)	21 (5 to 87)
Low value^a^	27.8	−19.3 (−21.0 to −17.5)^b^	91.1	−17.3 (−18.5 to −16.1)^b^	0	16 (3 to 63)
Point-of-care laboratory	37.7	−18.5 (−19.9 to −17.1)^b^	93.7	−16.3 (−17.3 to −15.3)^b^	0	15 (3 to 59)
Scheduled	26.8	−11.6 (−13.6 to −9.6)^b^	63.0	−6.2 (−8.9 to −3.4)^c^	62 (15 to 140)	86 (25 to 166)
High-value tests						
Cervical cancer screening^d^	23.3	−99.6 (−99.9 to −97.5)	88.7	10.6 (3.5 to 18.2)	0	0
HbA_1c_	59.9	−16.1 (−19.6 to −12.4)	94.9	−13.4 (−15.7 to −11.0)	0 (0 to 1)	15 (3 to 65)
Lipid panel	73.3	−15.3 (−18.2 to −12.2)	94.0	−14.7 (−17.0 to −12.3)	0 (0 to 1)	15 (3 to 58)
CRC screening	46.7	−14.4 (−21.7 to −6.3)	44.2	−4.1 (−16.1 to 9.6)	67 (14 to 183)	98 (15 to 178)
Mammography	30.5	−8.2 (−15.5 to −0.27)	84.1	−11.9 (−16.6 to −6.9)	51 (13 to 111)	74 (27 to 144)
Low-value tests						
Cervical cancer screening^d^	2.4	−97.4 (−99.6 to −81.1)	85.2	−100.0 (−100.0 to −100.0)	0	NA
Screening ECG^d^	2.1	−85.4 (−92.0 to −73.6)	72.2	−20.1 (−53.0 to 35.7)	0	12 (0 to 37)
Screening BMP	31.1	−18.5 (−24.5 to −11.9)	92.4	−18.0 (−21.9 to −14.0)	0	14 (3 to 49)
Screening LFT	12.4	−13.2 (−21.7 to −3.7)	91.6	−13.1 (−17.5 to −8.4)	0	16 (3 to 63)
Screening CMP	26.0	−22.9 (−29.5 to −15.8)	92.7	−18.9 (−23.2 to −14.4)	0	18 (4 to 64)
Screening CBC with or without differential	42.8	−18.0 (−22.2 to −13.7)	93.5	−18.1 (−20.8 to −15.3)	0	14 (3 to 55)
Prostate cancer screening	15.9	−2.0 (−33.7 to 44.7)	95.7	−13.8 (−27.5 to 2.6)	0 (0 to 7)	16 (4 to 45)
Thyroid screening	26.3	−20.8 (−26.7 to −14.5)	93.7	−18.0 (−21.7 to −14.2)	0	19 (3 to 62)
Screening urinalysis	5.4	−26.0 (−37.6 to −12.4)	92.9	−15.3 (−22.1 to −7.9)	0 (0 to 2)	17 (1 to 55)
CRC screening	12.1	−17.7 (−29.9 to −3.3)	44.0	8.7 (−9.4 to 30.3)	145 (76 to 212)	170 (113 to 253)

Abbreviations: BMP, basic metabolic panel; CBC, complete blood count; CMP, comprehensive metabolic panel; CRC, colorectal cancer; ECG, electrocardiogram; HbA_1c_, hemoglobin A_1c_; LFT, liver function test; NA, not applicable.

^a^ High-value and low-value composite categories did not include examination room tests (cervical cancer screening and screening ECG) given the small number of such tests ordered during virtual visits.

^b^ *P* < .001 after Holm-Bonferroni correction.

^c^ *P* < .05 after Holm-Bonferroni correction.

^d^ 1 Each of high-value and low-value cervical cancer screenings, and 11 low-value ECGs were ordered at virtual visits.

Median time to test completion was shorter for all tests (including scheduled tests) ordered during in-person visits vs virtual visits. For CRC screening, low-value tests had a longer median (IQR) time to completion than high-value tests (170 [113-253] days vs 98 [15-178] days). In multivariable survival analyses, adjusted HRs (AHRs) were significantly below 1 for all tests except for high-value cervical cancer screenings (which were almost never ordered virtually) and low-value CRC screenings (AHR, 1.3 [95% CI, 1.1-1.5] and 1.1 [95% CI, 0.84-1.4]), meaning that tests ordered at in-person visits were more likely to be completed at any given time point following an order (eTable 4 in Supplement 1). The AHRs were lower for low-value than high-value tests (0.37 [95% CI, 0.35-0.38] vs 0.51 [95% CI, 0.49-0.52]) and were much lower for laboratory than scheduled tests (0.34 [95% CI, 0.33-0.35] vs 0.86 [95% CI, 0.79-0.94]).

## Discussion

In this retrospective observational cohort study using EHR data across 87 primary care clinics to compare virtual and in-person annual visits by the same clinicians, we found that virtual visits were associated with lower rates of both test ordering (by clinicians) and test completion (by patients) than in-person visits. When comparing low-value with high-value tests, there was a slightly larger relative decrease for low-value tests from in-person to virtual visits for both test ordering and completion. When comparing laboratory with scheduled tests, there was a larger relative decrease for laboratory tests with virtual vs in-person visits for both test ordering and completion.

These results build on prior studies in various health systems and clinics showing fewer laboratory and imaging orders at virtual visits in general.^5,15,16,17,18,19^ For instance, studies before the COVID-19 pandemic^17^ or those focused on neck or back pain,^16^ cardiology,^19^ and otolaryngology^18^ found that virtual visits were associated with fewer test orders. Zeltzer et al^5^ reported that early in the pandemic in Israel, virtual visits had slightly less use of any laboratory testing than in-person visits. Our study advances that literature by finding that, beyond decreased test ordering, virtual visits were also associated with lower test completion on the order of 13.1% to 19.3%. This finding is echoed in a study in 2 primary care practices, which reported that fewer colonoscopies and cardiac stress tests were completed after virtual visits than in-person visits.^15^ Why completion rates are lower may vary by type of test. For laboratory tests, the reason may be the friction introduced by not already being in the office. We found lower rates of completion of scheduled mammograms yet no difference in completion of high- or low-value CRC screenings, which may reflect that these tests have different scheduling workflows. For instance, front-desk clinic staff might routinely help patients schedule their mammograms, but not their colonoscopies, when patients check out after in-person annual visits.

Our findings on low-value tests build on prior claims-based studies showing that Medicare patients in practices^20^ or health systems^3^ with greater telemedicine adoption were less likely to receive low-value tests, to demonstrate that clinicians are less likely to order these tests during virtual visits and patients are also less likely to complete them. While the magnitude of our findings on low-value testing was relatively modest, care cascades that may follow from initial testing have the potential to amplify the impact on total and out-of-pocket spending.^10,11,26^ However, we found a similar, and concerning, pattern for several high-value tests, in contrast to a prior study finding that Medicare patients in health systems with greater vs lesser telemedicine adoption received high-value breast, colorectal, and cervical cancer screening tests at equivalent rates.^4^ This result is especially important given suboptimal rates of high-value testing demonstrated in both studies and in many other US settings.^32,33,34,35,36,37^ It suggests that telemedicine is a somewhat blunt instrument for reducing testing for both clinicians and patients. More optimistically, it may reflect the possibility that, to some extent, these high-value services—which often appear as care gaps that clinicians are prompted to complete when they are viewing the patient’s medical record—may be ordered outside of the context of the annual virtual visit.

The results of this study suggest an opportunity to harness the potential benefits of the virtual modality in reducing low-value testing while protecting against its potential to diminish high-value test ordering and completion. There may be a role for interventions aimed at clinicians and patients to better address care gaps during virtual visits, such as through formalized front-desk follow-up after virtual visits to help patients schedule or otherwise facilitate completion of high-value tests.^15^ As care delivery models evolve, further work is needed to understand the extent to which high-value tests are, or can be, completed outside of these visits (eg, prompted by patient portal reminders).^38,39^ These results also highlight the importance of appropriate clinical indications for virtual visits within the context of primary care relationships.^40^

### Strengths and Limitations

The strengths of this study include the use of detailed EHR data, exact-matching visits on clinician and visit year to control for differences in practice style^30^ and temporal trends, and propensity score matching on key factors, such as patients’ distance to clinic and prior portal engagement. The study also has limitations. There is uncertain generalizability beyond the large MGB health system, which serves a predominantly White, English-speaking patient population, or beyond annual visits. We acknowledge the potential misclassification of tests as high or low value due to clinician billing inconsistencies. Although we accounted for many potential confounders and specified visits eligible or relevant for a given high- or low-value test, there was likely residual bias by indication in patients and clinicians choosing to schedule a virtual visit vs an in-person visit. For example, patients who select a virtual (vs in-person) annual visit may also have less desire or ability to follow through with ordered tests in ways that study variables do not capture. Finally, we did not assess clinical outcomes associated with test ordering and completion. Future research could examine other visit types and health care settings and explore effective ways to selectively promote high-value testing.

## Conclusions

In this cohort study, telemedicine was associated both with less ordering and less completion of both low- and high-value tests, with slightly larger reductions in low-value testing and point-of-care laboratory tests. These results suggest that, while telemedicine may have many advantages, including potential spillover benefits for low-value testing, it should be balanced with targeted and complementary efforts to ensure completion of high-value care.

## Notes

Supplement 1. **eMethods.** Supplemental Definitions and Methods **eTable 1.** Procedure Codes and Labels Used to Identify Tests **eTable 2.** Definitions of High- and Low-Value Tests **eFigure.** Consort Diagram of Sample Creation **eTable 3.** Ordering and Completion of Tests for In-person and Virtual Visits, by Test Category **eTable 4.** Adjusted Hazard Ratio for Time to Test Completion

Supplement 2.
Data Sharing Statement
