# Supplementary material for: Test Ordering and Completion During Virtual vs In-Person Annual Visits
Source: JAMA Netw Open. 2026 Feb 25;9(2):e260013. doi: 10.1001/jamanetworkopen.2026.0013 (PMC12936881; doi:10.1001/jamanetworkopen.2026.0013)
Supplement: Supplement 1. — eMethods. Supplemental Definitions and Methods eTable 1. Procedure Codes and Labels Used to Identify Tests eTable 2. Definitions of High- and Low-Value Tests eFigure. Consort Diagram of Sample Creation eTable 3. Ordering and Completion of Tests for In-person and Virtual Visits, by Test Category eTable 4. Adjusted Hazard Ratio for Time to Test Completion [file jamanetwopen-e260013-s001.pdf]

Supplemental Online Content

Ganguli I, Daley NE, Mehrotra A, Rosenthal MB, Cutler DM. Test ordering and completion during virtual vs in-person annual visits. *JAMA Netw Open*. 2026;9(2):e260013. doi:10.1001/jamanetworkopen.2026.0013

eMethods. Supplemental Definitions and Methods

eTable 1. Procedure Codes and Labels Used to Identify Tests

eTable 2. Definitions of High- and Low-Value Tests

eFigure. Consort Diagram of Sample Creation

eTable 3. Ordering and Completion of Tests for In-person and Virtual Visits, by Test Category

eTable 4. Adjusted Hazard Ratio for Time to Test Completion

This supplemental material has been provided by the authors to give readers additional information about their work.

© 2026 Ganguli I et al. *JAMA Network Open*

eMethods. Supplemental Definitions and Methods

Iterative chart review –To confirm validity of the data extract elements and outcome measures, we underwent a cyclical process of checking data extract elements against the patients' electronic health records to refine operational definitions or abstraction rules as needed based on identified ambiguities and inconsistencies, confirm data accuracy and validity, and ensure consistent abstraction. In one example, we pulled a random sample of 25-30 encounters to verify that the visit text labels we were using to designate visit modality aligned with information in the visit notes and billing codes used for the visit. In another example, when validating our extraction of CRC screen ordering and completion data, we examined a random sample of visits and reviewed notes and test histories to check for CRC orders we may have missed. We found that orders that were not completed after some time were marked as canceled in the EHR, such that they were missed in our data pull. So we changed extraction criteria for all tests to include orders that were labeled "cancelled" if they met certain additional criteria based on cancellation reason and time of cancellation relative to test order (see below), and did additional chart review to verify that this new approach captured ordered and completed tests as intended.

Annual visit text labels - 'Annual', 'Annual Wellness', 'Annual Wellness Initial', 'Annual Wellness MCR Initial', 'Annual Wellness MCR Subsequent', 'Annual Wellness Subsequent', 'Complete Physical Exam', 'Physical', 'Wellness Initial', 'Wellness Return', 'VIRTUAL ANNUAL WELLNESS VISIT','VIRTUAL PHYSICAL'. Virtual annuals included video and audio visits.

Combining complete blood counts (CBC) with and without differential – We separately pulled orders and completions for CBC with differential and CBC without differential and then created a combined measure that counted an order or completion of either test, choosing the earliest possible dates for the order and completion.

Identifying completion of mammography and colorectal cancer screening – For colorectal cancer screening and mammography, for which clear order-to-completion linkages were not available in the electronic health record (EHR), we identified if the service was completed within 11 months of the original order and took the date of the first such instance.

Refining test completion definition – Because orders that were not completed may have expired and then appeared as canceled in the data, orders that were placed then canceled before completion were counted as ordered but not completed unless canceled within 24 hours of the order or with one of the following listed reasons for cancellation: "Entered in Error", "Exam No Longer Needed Per Care Team", "Original order incorrect as discussed with care team", "Ordered on Incorrect Episode/Contact", or "Ordered on Wrong Patient." If a patient had another order in the same category as a canceled order, we used the non-canceled order.

Combining metabolic panels in binomial models – The basic metabolic panel (BMP), liver function test panel (LFT), and complete metabolic panel (CMP) are 3 laboratory test panels that can be ordered individually yet are interrelated (a BMP with an LFT is equivalent to a CMP). When analyzing testing patterns across all low-value tests or all laboratory tests, we accounted for this non-independence by treating each CMP as a BMP plus LFT. In these models, visits eligible for both BMP and LFT (meaning they were eligible for CMP) were counted as two visits; orders of CMP were counted as two orders, as if they were a BMP order and an LFT order, and completions of CMP were counted as two completions.

© 2026 Ganguli I et al. *JAMA Network Open*

eTable 1. Procedure Codes and Labels Used to Identify Tests

EHR Procedure Code[a]	Procedure Label
**Cervical Cancer Screening**	
444394	PAP SMEAR
444394	PAP TEST
125307	PAP AND HPV W/ GENOTYPING
546207	THINPREP TIS PAP AND HPV MRNA E6/E7 REFLExHPV 16,18/45
535813	GENETIC PROBE AMPLIFICATION FOR HUMAN PAPILLOMAVIRUS
125309	PAP WITH REFLExTO HPV
227071	GENETIC PROBE AMPLIFICATION FOR HUMAN PAPILLOMAVIRUS
502817	THINPREP PAP & HPV
546221	THINPREP TIS PAP REFLExHPV MRNA E6/E7, CHLAMYDIA/N.GONORRHOEAE
546229	THINPREP TIS PAP, HPV MRNA E6/E7 RFxHPV 16,18/45, CHLAMYDIA/N.GONORRHOEAE
546205	THINPREP TIS PAP (REFL) HPV MRNA E6/E7
546175	THINPREP PAP AND HPV MRNA E6/E7
554684	HUMAN PAPILLOMAVIRUS (HPV) SCREENING
125311	PAP ONLY
554684	HUMAN PAPILLOMAVIRUS
546177	THINPREP PAP AND HPV MRNA E6/E7 REFLExHPV 16,18/45
548619	PAP SMEAR
546185	THINPREP PAP AND HR HPV DNA REFLExGENOTYPES 16,18
546183	THINPREP PAP AND HR HPV DNA
502821	THINPREP PAP & HPV MRNA E6/E7
546203	THINPREP TIS PAP
546219	THINPREP TIS PAP REFLExHPV MRNA E6/E7
546189	THINPREP PAP REFLExHPV MRNA E6/E7
546173	THINPREP PAP (REFL) HPV MRNA E6/E7
546179	THINPREP PAP AND HPV MRNA E6/E7, CHLAMYDIA/N.GONORRHOEAE
546191	THINPREP PAP REFLExHPV MRNA E6/E7, CHLAMYDIA/N.GONORRHOEAE
546209	THINPREP TIS PAP AND HPV MRNA E6/E7, CHLAMYDIA/N.GONORRHOEAE
546171	THINPREP PAP
546223	THINPREP TIS PAP REFLExHPV MRNA E6/E7, CT/NG, TRICH
546147	SUREPATH IMAGING PAP AND HPV DNA
546195	THINPREP PAP RFxHR HPV
546197	THINPREP PAP RFxHR HPV DNA AND C. TRACHOMATIS AND N. GONORRHOEAE
546211	THINPREP TIS PAP AND HPV MRNA E6/E7, CT/NG, TRICH
546213	THINPREP TIS PAP AND HR HPV DNA
546199	THINPREP PAP, HPV MRNA E6/E7 RFxHPV 16,18/45, CHLAMYDIA/N.GONORRHOEAE
546181	THINPREP PAP AND HPV MRNA E6/E7, CT/NG, TRICH
555114	THINPREP PAP AND HR HPV DNA
**Hemoglobin A1c testing**	
90	HEMOGLOBIN A1C
812	GLUCOSE
535086	GLUCOSE, FASTING
1610	POCT GLUCOSE

© 2026 Ganguli I et al. *JAMA Network Open*

579055	POCT GLUCOSE
175090	GLUCOSE TOLERANCE TEST, 2 HR
535931	GLUCOSE, PLASMA
569500	GLUCOSE TOLERANCE TEST, 2 HOUR
175089	GLUCOSE TOLERANCE TEST, 3 HR
400000087	OUTSIDE GLUCOSE TOLERANCE
493897	GLUCOSE TOLERANCE TEST, 1 HR
**Lipid Testing**	
684	LIPID PANEL
494137	LIPID PROFILE WITH DIRECT LDL
56932	POCT LIPID PANEL
545387	LIPID PANEL WITH REFLExTO DIRECT LDL
112060	LIPID PANEL WITH REFLExTO DIRECT LDL
555649	LIPID PANEL WITH REFLExTO DIRECT LDL
538323	LIPID PANEL
578849	LIPID PROFILE
535786	LIPID PANEL
684	LIPID PANEL
**Colorectal Cancer Screening**	
7428201	CASE REQUEST GI REFERRING (ENDOSCOPY, EGD, GASTRO, COLONOSCOPY, BREATH TEST)
553309	COLOGUARD (ExCT SCIENCES)
74282	CASE REQUEST GI (ENDOSCOPY, EGD, GASTRO, COLONOSCOPY, BREATH TEST)
2102001016	AMB REFERRAL TO NS COLONOSCOPY
543387	FECAL IMMUNOCHEMICAL TEST x (FIT)
543389	FECAL IMMUNOCHEMICAL TEST x (FIT)
227045	FECAL OCCULT BLOOD
495169	FECAL OCCULT BLOOD, MULTIPLE
550718	FECAL IMMUNOCHEMICAL TEST x2 (FIT)
142317	CT ABDOMEN/PELVIS COLONOGRAPHY (SCREENING)
560784	COLONOSCOPY-NON-INTERFACED
225276	FECAL OCCULT BLOOD TEST 3
142317	CT ABDOMEN/PELVIS COLONOGRAPHY (SCREENING)
573295	POCT IFOBT CANCER SCREEN
225274	FECAL OCCULT BLOOD TEST 1
543387	FECAL IMMUNOCHEMICAL BLOOD TEST x (FIT)
1414	ENDOSCOPY, SIGMOID
577727	FECAL IMMUNOCHEMICAL TEST x (FIT)
578216	FECAL GLOBIN & IMMUNOCHEMISTRY TEST
220983	RIGID/FLExSIGMOIDOSCOPY
1398	ENDOSCOPY, COLON
7428201	CASE REQUEST GI REFERRING (ENDOSCOPY, EGD, GASTRO, COLONOSCOPY, BREATH TEST)
**Mammography**	
135739	BI MAMMOGRAM SCREENING (BILATERAL)
134633	BI MAMMOGRAM DIAGNOSTIC (BILATERAL)
**Electrocardiogram**	
509	ECG 12-LEAD

**Basic Metabolic Panel**	
678	BASIC METABOLIC PANEL
507579	BMP/RFP
535599	BASIC METABOLIC PANEL
571841	BASIC METABOLIC 7 (WITHOUT CALCIUM)
56914	POCT BASIC METABOLIC PANEL
535253	BASIC METABOLIC PANEL
**Liver Function Testing**	
133917	LFTS (HEPATIC PANEL)
535639	HEPATIC FUNCTION PANEL
545365	HEPATIC FUNCT PANEL W/O TP
**Comprehensive Metabolic Panel**	
174816	COMPREHENSIVE METABOLIC PANEL
477761	CMP & LFT
477765	CMP & RFP
535609	COMPREHENSIVE METABOLIC PANEL
538299	COMPREHENSIVE METABOLIC PANEL
**Complete Blood Count +/- Differential**	
1696	CBC AND DIFFERENTIAL
498357	CBC WITH MANUAL DIFFERENTIAL
535605	CBC AND DIFFERENTIAL
557189	CBC WITH AUTOMATED DIFFERENTIAL
552320	CBC W/MANUAL DIFFERENTIAL
568174	CBC WITH DIFF
546811	CBC, INCLUDES DIFF/PLT (QUEST)
1698	CBC
111208	CBC W/O DIFF.
538293	CBC
538993	CBC
571863	CBC, NO DIFFERENTIAL/PLATELET
**Prostate Specific Antigen Testing**	
880	PSA (SCREENING)
248889	PSA
174913	PSA, FREE AND TOTAL
545435	PSA, TOTAL WITH REFLExTO PSA, FREE
572063	PSA (REFLExTO FREE) (SERIAL)
548739	PSA, TOTAL
548739	PSA, TOTAL (QUEST)
538331	PSA
**Thyroid Stimulating Hormone Testing**	
133922	TSH WITH REFLEX
906	TSH
563945	TSH WITH CASCADE
519199	THYROID MONITORING PANEL
567579	TSH (REFL)
546241	TSH W/REFLExTO FT4
507363	THYROID PANEL WITH TSH
548917	TSH (QUEST)

© 2026 Ganguli I et al. *JAMA Network Open*

538351	TSH
559882	THYROID PROFILE (TSH, FREE T4)
572133	TSH RFxON ABNORMAL TO FREE T4
507609	THYROID PROFILE
108448	TSH WITH REFLEX
**Urinalysis**	
1804	URINALYSIS
519209	URINALYSIS W/REFLExURINE CULTURE
214538	URINALYSIS WITH SEDIMENT
564052	URINALYSIS W/MICROSCOPY
564051	URINALYSIS W/O MICROSCOPY
553156	URINALYSIS
535317	URINALYSIS
535795	URINALYSIS
551282	URINALYSIS W/MICROSCOPY
138439	URINALYSIS
552259	URINALYSIS REFLEX
536527	URINALYSIS
550072	URINALYSIS, DIPSTICK ONLY
138435	URINALYSIS DIPSTICK
535759	URINALYSIS

[a] EHR procedure codes are the unique service identifiers used in the Mass General Brigham EHR.

© 2026 Ganguli I et al. *JAMA Network Open*

eTable 2. Definitions of High- and Low-Value Tests

Test	Categories	Visit eligibility criteria	Relevant Inclusion/Exclusion Details	Relevant Health Maintenance Modifiers
High-value cervical cancer screening	High-Value Tests; Exam Room Tests	Patient visits in which patient is 1) female AND 2) a) between 21-65 AND NO prior Pap smear in the designated interval (determined by HM modifier) OR b) has cervical cancer risk-related diagnosis on problem list OR as visit diagnosis OR HM modifier 80,156, or 177 AND 3) NO "not a candidate" HM modifier	*Inclusion* – Cervical cancer-related diagnoses ICD-10 Codes: C53*, N87*, D06*, Z86.001-002, R87.6*, R87.81*, R87.82*, Z85.40-44, C57.9, C51*-53*	NC: "Advanced Dementia"<br>NC: "Terminal Illness"<br>NC: "Comfort care only"<br>NC: "Not a candidate for Pap Smear"<br>NC: "Not a candidate for cervical cancer screening"<br>80: "LSIL pap with negative HPV age 25+; 1 year pap with reflex HPV or colposcopy recommended"<br>156: "Normal Pap with positive HPV; 1 year pap with HPV cotest or colposcopy recommended"<br>177: "Low grade cervical abnormality; 1 year pap with reflex HPV recommended"
High-value Hemoglobin A1c	High-Value Tests; Lab Tests	Patient visits in which patient is 1) a) in Diabetes Registry OR b) Has "prediabetes": prediabetes on problem list OR prior Hemoglobin A1c >5.6 in past three years OR c) Age 35-≤70 AND BMI>25 AND no HgbA1c in 2 years before visit, OR d) There IS modifier 66 or 185 AND 2) No "not a candidate" HM modifiers	*Inclusion* – Prediabetes ICD-10 Codes: R73.03	NC: "Advanced Dementia"<br>NC: "Terminal Illness"<br>NC: "Comfort care only"<br>NC: "Not a candidate for routine hemoglobin A1C testing"<br>NC: "Not a candidate for diabetes screening"<br>66: "Hemoglobin A1C level every 6 months"<br>185: "Hemoglobin A1C every 12 months"

High-value lipid panel	High-Value Tests; Lab Tests	Patient visits in which 1) a) Hyperlipidemia on problem list at time of visit OR listed as visit diagnosis OR b) Statin/cholesterol med on drug list OR c) patients in CVD registry OR d) Hypertension registry OR e) Diabetes registry, OR f) patient 40-75 AND no lipid test in past 4 years (OR 1 year if HM modifier 32), AND 2) No "not a candidate" HM modifiers	*Inclusion* – Hyperlipidemia ICD-10 Codes: E78.0, E78.00, E78.01, E78.1, E78.2, E78.3, E78.4, E78.41, E78.49, E78.5<br>Cholesterol Medications defined as medications labeled with pharmaceutical class "antihyperlipidemic" in the EHR	NC: "Advanced Dementia"<br>NC: "Terminal Illness"<br>NC: "Comfort care only"<br>NC: "Not a candidate for routine lipid testing"<br>32: "Lipid Panel Every 2 Years"
High-value colorectal cancer screening	High-Value Tests; Later-Scheduled Tests	Patient visits in which patient is 1) 45-75 years old AND 2) No CRC screening in prior time (no Stool DNA-FIT in past 2 years; no CT colonography or flexible sigmoidoscopy in past 4 years; no colonoscopy in past 9 years, or one year less than the timeframe outlined in HM modifiers 35, 58, 95, 113, 124, 134, 152, 167) AND 3) No "not a candidate" HM modifier	None	NC: "Advanced Dementia"<br>NC: "Terminal Illness"<br>NC: "Comfort care only"<br>NC: "Not a candidate for colon cancer screening"<br>35: "Colonoscopy every 5 years"<br>58: "Colonoscopy every 10 years"<br>95: "Colonoscopy every 7 years"<br>113: "Colonoscopy every 3 years"<br>124: "Colonoscopy every 6 months"<br>134: "Colonoscopy every 2 years"<br>152: "Colonoscopy every 3 months"<br>167: "Colonoscopy every 1 year"
High-value mammography	High-Value Tests; Later-Scheduled Tests	Patient visits in which patient is 1) female AND 2) a) 40-74 years old AND b) NO bilateral mastectomy (on med history, surg history, or problem list) OR 3) Has breast cancer in problem list or visit diagnoses AND 4) No "not a candidate" HM modifier	*Exclusion* – Mastectomy ICD-10 codes: Z90.1*. Mastectomy CPT codes: 19301-19306; ICD-10 codes for breast cancer: C50.011, C50.012, C50.019, C50.021, C50.022, C50.029, C50.111, C50.112, C50.119, C50.121, C50.122, C50.129, C50.211, C50.212, C50.219, C50.221, C50.222, C50.229, C50.311, C50.312, C50.319, C50.321, C50.322, C50.329, C50.411, C50.412, C50.419, C50.421, C50.422, C50.429, C50.511, C50.512, C50.519, C50.521, C50.522, C50.529, C50.611, C50.612, C50.619, C50.621, C50.622, C50.629, C50.811, C50.812, C50.819, C50.821, C50.822, C50.829, C50.911, C50.912, C50.919, C50.921,	NC: "Advanced Dementia"<br>NC: "Terminal Illness"<br>NC: "Comfort care only"<br>NC: "Not a candidate for breast cancer screening"

			C50.922, C50.929, D05.00, D05.01, D05.02, D05.10, D05.11, D05.12, D05.80, D05.81, D05.82, D05.90, D05.91, D05.92, Z17.0, Z17.1, Z19.1, Z19.2, Z85.3, Z86.000	
Low-value cervical cancer screening	Low-Value Tests; Exam Room Tests	Patient visits in which patient is 1) Female AND 2) a) Age <21 or >65yo OR there was prior Pap smear in designated interval (determined by HM modifier) AND b) NO "Cervical Cancer" related diagnosis on problem list OR visit diagnosis, OR 3) there IS a "not a candidate" modifier	*Exclusion* – Cervical cancer-related diagnoses ICD-10 Codes: C53*, N87*, D06*, Z86.001-002, R87.6*, R87.81*, R87.82*, Z85.40-44, C57.9, C51*-53*	NC: "Advanced Dementia"<br>NC: "Terminal Illness"<br>NC: "Comfort care only"<br>NC: "Not a candidate for Pap Smear"<br>NC: "Not a candidate for cervical cancer screening"<br>5: "Pap smear every 5 years"<br>80: "LSIL pap with negative HPV age 25+; 1 year pap with reflex HPV or colposcopy recommended"<br>156: "Normal Pap with positive HPV; 1 year pap with HPV cotest or colposcopy recommended"<br>177: "Low grade cervical abnormality; 1 year pap with reflex HPV recommended"
Low-value screening electrocardiogram	Low-Value Tests; Exam Room Tests	Patient visits in which 1) patient is NOT on CVD registry AND there are no relevant symptoms in the visit diagnoses OR 2) there is a "not a candidate" HM modifier	*Exclusion* – Relevant symptom ICD-10 codes: R00.0, R00.2 (Tachycardia, palpitations); R06.0*, R06.2-4, R06.89, R06.9 (Dyspnea); R07.1, R07.2, R07.8*, R07.9 (Chest pain); R11, R40.0-1, R55 (Unconsciousness/syncope); R42 (Dizziness); R60 (Lower extremity edema)	NC: "Advanced Dementia"<br>NC: "Terminal Illness"<br>NC: "Comfort care only"
Low-value screening basic metabolic panel	Low-Value Tests; Lab Tests	Patient visits in which patient is NOT on hypertension, diabetes, or CKD registry AND NO modifier 56, 60, 61, 65, 128, 193 AND no relevant symptoms in the visit diagnoses	*Exclusion* – Relevant symptom ICD-10 codes: N23, R10* (Flank pain/renal colic); N13.0-2, N13.3*, N20*, N21*, N22* (Nephrolithiasis, ureteral calculus); E15, E16.0-2, E20.0, E20.8-9, E21.0-3, E83.51, E83.52, E87.0, E87.1, E87.5-6, E87.8, E89.1-2, R73.03, R73.09, R73.9, T38.X* (Electrolyte/fluid disorders); E40, E41, E42, E43, E44*, E46, E64.0, K91.2, T74.01* (Malnutrition)	NC: "Advanced Dementia"<br>NC: "Terminal Illness"<br>NC: "Comfort care only"<br>56: "Creatinine Level every 6 months"<br>60: "Creatinine Level annually"<br>61: "Potassium level annually"<br>65: "Chronic Kidney Disease"<br>128: "Potassium level every 6 months"<br>193: "Creatinine Level every 3 months"

© 2026 Ganguli I et al. *JAMA Network Open*

Low-value screening liver function test	Low-Value Tests; Lab Tests	Patient vistis in which 1) patient does NOT have liver disease on problem list AND NO modifier 90, 183, 161 AND no relevant symptoms in the visit diagnoses OR 2) there is a “not a candidate” HM modifier	*Exclusion* – Liver disease ICD-10 codes: K70*-K76*<br>Relevant symptoms ICD-10 codes: K70*-K76* (Liver disease); K80* (Biliary colic, gallstones); R10* (Abdominal pain); E40, E41, E42, E43, E44*, E46, E64.0, K91.2, T74.01* (Malnutrition)	NC: “Advanced Dementia”<br>NC: “Terminal Illness”<br>NC: “Comfort care only”<br>90: “ALT level annually”<br>183: “Alkaline Phosphatase level annually”<br>161: “ALT level every 6 months”
Low-value screening comprehensive metabolic panel	Low-Value Tests; Lab Tests	Patient visits in which 1) patient is NOT on hypertension, diabetes, or CKD registry AND does NOT have liver disease on problem list AND NO modifier 56,60,61,65,128,193,90,183,161 AND no relevant symptoms in the visit diagnoses OR 2) there is a “not a candidate” HM modifier	*Exclusion* – Liver disease ICD-10 codes: K70*-K76*<br>Relevant symptom ICD-10 codes: K70*-K76* (Liver disease); K80* (Biliary colic, gallstones); R10* (Abdominal pain); N23, R10* (Flank pain/renal colic); N13.0-2, N13.3*, N20*, N21*, N22* (Nephrolithiasis, ureteral calculus); E15, E16.0-2, E20.0, E20.8-9, E21.0-3, E83.51, E83.52, E87.0, E87.1, E87.5-6, E87.8, E89.1-2, R73.03, R73.09, R73.9, T38.X* (Electrolyte/fluid disorders); E40, E41, E42, E43, E44*, E46, E64.0, K91.2, T74.01* (Malnutrition)	NC: “Advanced Dementia”<br>NC: “Terminal Illness”<br>NC: “Comfort care only”<br>56: “Creatinine Level every 6 months”<br>60: “Creatinine Level annually”<br>61: “Potassium level annually”<br>65: “Chronic Kidney Disease”<br>128: “Potassium level every 6 months”<br>193: “Creatinine Level every 3 months”<br>90: “ALT level annually”<br>183: “Alkaline Phosphatase level annually”<br>161: “ALT level every 6 months”
Low-value screening complete blood count +/- differential	Low-Value Tests; Lab Tests	Patient visits in which patient is 1) NOT on CVD registry AND 2) no relevant symptoms in the visit diagnoses AND 3) no CBC justification HM modifier OR 4) there is a “not a candidate” HM modifier	*Exclusion* – Relevant symptom ICD-10 codes: C90.1*, C91*-C95* (Leukemia); C94.6, D46*, D47.4, D50*-D53*, D55*-D56*, D57.00-3, D57.9, D57.1, D57.211-3, D57.218-9, D57.3, D57.40, D57.411-3, D57.418-9, D57.42, D57.431-3, D57.438-9, D57.44, D57.451-3, D57.458-9, D57.80-811-3, D57.818-9, D58*-60*, D61.01, D61.09, D61.1-D61.9, D63*-D64*, D64*, D75.81 (Anemia); A*, B* (Infections); D57*, D78.0*, D78.2*, D78.31-2, E36.0*, E89.81*, E89.820-1, G97.3*, G97.5*, G97.61-2, H59.11*, H59.12*, H59.31*, H59.32*, H59.33*, H59.34*, H95.2*, H95.4*, H95.51-2, I97.4*, I97.61*, I97.63*, I97.620-1, I97.81*, I97.82*, J95.6*, J95.83*, J95.860-1, K91.6*, K91.84*,	NC: “Advanced Dementia”<br>NC: “Terminal Illness”<br>NC: “Comfort care only”<br>“CBC level every 3 months”<br>“CBC level annually”<br>“CBC level every 6 months”

© 2026 Ganguli I et al. *JAMA Network Open*

			K91.70-1, K92.2, L76.0*, L76.2*, L76.31-2, M96.81*, M96.83*, M96.840-1, N99.6*, N99.82*, N99.840-1, R04*, R23.3, R58 (Hematemesis, melena, internal bleeding, petechiae, epistaxis, ecchymoses); D47.3, D69.3, D69.4*, D69.6, D75.82, M31.1* (Thrombocytosis, ITP, Thrombocytopenia); D70*, D73.81 (Neutropenia); R10* (Abdominal pain); O86.4, R50.2, R50.9, T50.905* (Fever); R63.4 (Unintended weight loss)	
Low-value prostate cancer screening	Low-Value Tests; Lab Tests	Patient visits in which patient is 1) Male AND 2) a) No "Prostate Cancer" on problem list or visit diagnosis AND b) Age >70 OR 3) there is a "not a candidate" HM modifier	*Exclusion* – Prostate Cancer ICD-10 Codes: C61, D07.5, Z85.46	NC: “Advanced Dementia”<br>NC: “Terminal Illness”<br>NC: “Comfort care only”
Low-value thyroid screening	Low-Value Tests; Lab Tests	Patient visits in which 1) patient does NOT have thyroid-related problem on problem list AND no relevant symptoms in the visit diagnoses OR 2) there is a “not a candidate” HM modifier	*Exclusion* – Thyroid related problem ICD-10 codes: (ICD10: E00*, E01.8, E02, E03.0-4*, E03.8-9, E89.0 (Hypothyroidism); E01.0-E01.2, E05*-E07* (Thyrotoxicosis, hyperthyroidism, Graves’ disease, thyroiditis, unspecified disorder of thyroid); C73, C79.89, D09.3, D34, D44.0, D44.9, E31.20, Z80.8, Z83.41, Z85.850 (Thyroid neoplasm, personal/family history of multiple endocrine neoplasia syndrome); Q90* (Down’s syndrome)<br>Relevant symptom ICD-10 codes: M62.81, R53*, M79.1*; H05.2*, G23.1, H49.3*-4*, H51.2*, I47*, R00*, K59.0*, L74.5*, R61, R13.1*, R22.1, R49.0, R63.4-5, R94.6, R68.89 (Muscle weakness, myalgia, fatigue, exophalmos, ophthalmoplegia, Tachycardia, bradycardia, palpitations, Constipation, Excessive sweating, Neck lump, dysphonia, dysphagia, Abnormal/unexpected weight gain/loss, Abnormal results of thyroid function studies, Heat or cold intolerance)	NC: “Advanced Dementia”<br>NC: “Terminal Illness”<br>NC: “Comfort care only”

© 2026 Ganguli I et al. *JAMA Network Open*

Low-value screening urinalysis	Low-Value Tests; Lab Tests	All patient visits without relevant symptoms in the visit diagnoses OR there is a “not a candidate” HM modifier	*Exclusion* – Relevant symptoms ICD-10 codes: S37.0*, N17*, N19* (Acute kidney injury/failure); N40*, N42.83 (Benign prostatic hyperplasia); N23, R10* (Flank pain/renal colic); N10*, N11*, N12, N13.6, N30*, N39.0, B37.4* (Urinary tract infection); R30.0, R30.9, R31*, R35*, R36.1, R39.11, R39.15, R80.0-1, R80.3-8, R82.7*, R82.8*, R82.9* (Hematuria, hematospermia, dysuria, urinary frequency, excessive urination, nocturia, urinary urgency, hesitancy, pus in urine, proteinuria); A56.1*, N24.1-2, N41*, N45.1-3, N70*-N77* (Urethritis, prostatitis, epididymitis, pelvic infection)	NC: “Advanced Dementia”<br>NC: “Terminal Illness”<br>NC: “Comfort care only”
Low-value colorectal cancer screening	Low-Value Tests; Later-Scheduled Tests	Patient visits in which patient is 1) a) >85 years old OR there was prior CRC screen in designated interval (determined by HM modifier) AND b) No "Colorectal Cancer" on problem list or visit diagnosis OR 2) there is a "not a candidate" modifier	*Exclusion* – Colorectal cancer ICD-10 Codes: C18-C20, C49.A4, C49.A5, D01.0-2, Z85.030, Z85.038, Z85.040, Z85.048	NC: “Advanced Dementia”<br>NC: “Terminal Illness”<br>NC: “Comfort care only”<br>NC: “Not a candidate for colon cancer screening”

HM = Health Maintenance; ICD-10 = International Classification of Diseases, 10th Revision; NC = Not a Candidate; LSIL = Low-grade Squamous Intraepithelial Lesion; HPV = Human Papillomavirus; CKD = Chronic Kidney Disease; ALT = Alanine Aminotransferase; CVD = Cardiovascular Disease

eFigure. Consort Diagram of Sample Creation

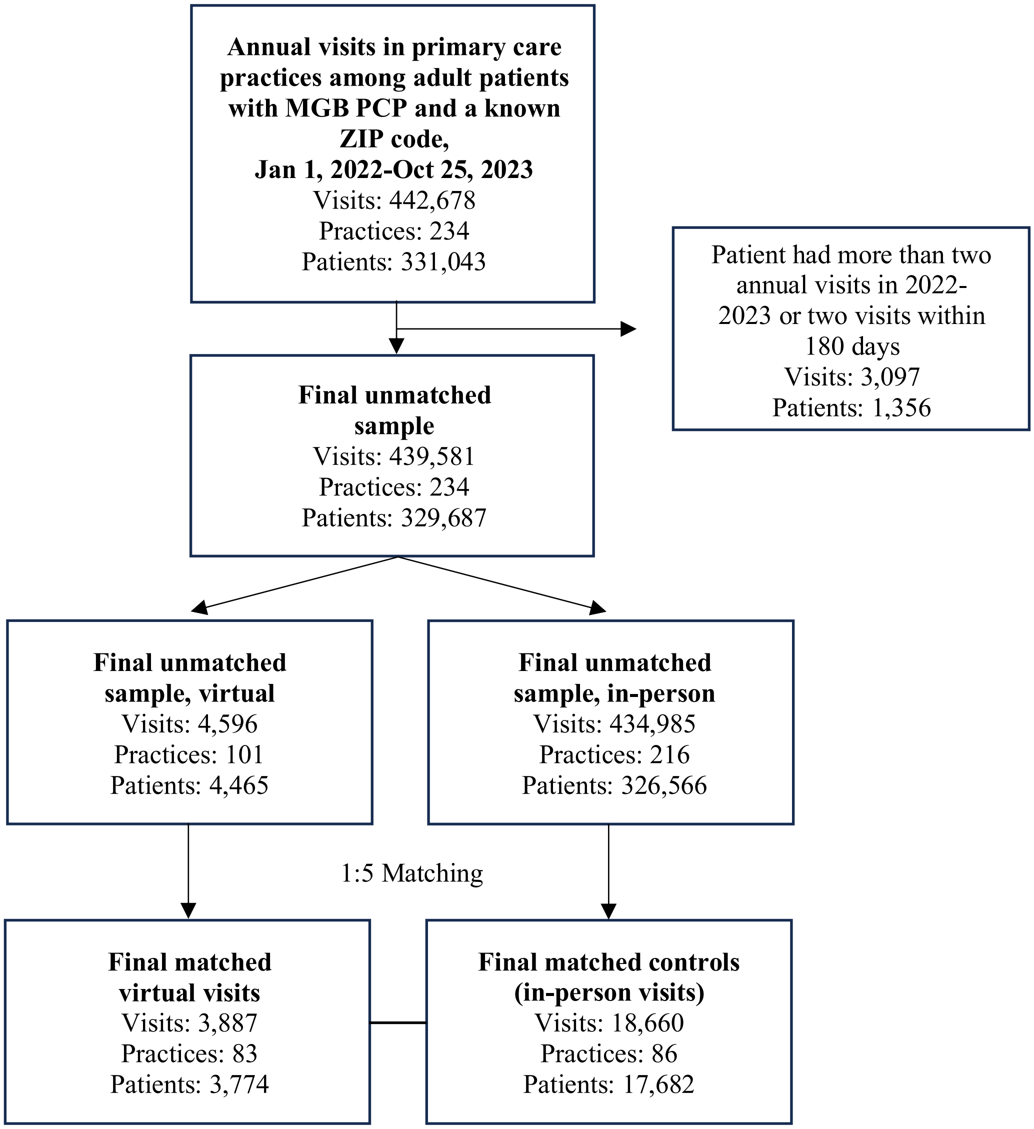

© 2026 Ganguli I et al. *JAMA Network Open*

eTable 3. Ordering and Completion of Tests for In-person and Virtual Visits, by Test Category

**Test**	Eligible patient-visits, in-person	Eligible patient-visits, virtual	Percent of eligible visits with a test order, in-person	Percent of eligible visits with a test order, virtual	Adjusted percentage point difference in orders per visit, virtual minus in-person; pp (95% CI)	Percent of visits with a test completion, in-person	Percent of visits with a test completion, virtual	Percent of visits with a test completion (contingent on ordering), in-person	Percent of visits with a test completion (contingent on ordering), virtual	Adjusted percentage point difference in completion rate (contingent on ordering), virtual minus in-person; pp (95% CI)
**Composite Test Categories**										
High-value[a]	34,300	7,105	54.8	46.7	-8.3 (-9.7, -6.9)	48.1	35.7	87.7	76.5	-11.5 (-13.3, -9.6)
Low-value[a]	82,764	17,126	27.8	22.4	-5.7 (-6.9, -4.6)	25.4	16.9	91.1	75.3	-15.8 (-18.0, -13.5)
Point-of-care laboratory	96,258	20,078	37.7	30.8	-7.5 (-8.7, -6.2)	35.3	24.2	93.7	78.5	-15.3 (-17.2, -13.5)
Scheduled	20,806	4,153	26.8	23.2	-3.3 (-4.5, -2.1)	16.9	13.7	63.0	59.1	-3.9 (-7.1, -0.49)
**High-Value Tests**										
Cervical cancer screening[b]	6,123	1,173	23.3	0.09	-23.3 (-24.4, -22.2)	20.6	0.09	88.7	100.0	9.4 (3.0, 15.9)
HbA_{1c}	11,389	2,402	59.9	50.1	-9.6 (-11.8, -7.4)	56.8	41.3	94.9	82.3	-12.7 (-14.9, -10.4)
Lipid panel	10,035	2,128	73.3	61.4	-11.1 (-13.4, -8.9)	68.9	49.3	94.0	80.3	-13.9 (-16.1, -11.6)
CRC screening	4,264	844	46.7	38.9	-6.7 (-10.3, -3.0)	20.7	16.6	44.2	40.2	-1.8 (-7.5, 3.9)
Mammography	8,612	1,731	30.5	27.6	-2.5 (-4.8, -0.15)	25.7	20.5	84.1	74.3	-10.0 (-14.2, -5.8)
**Low-Value Tests**										
Cervical cancer screening[b]	6,853	1,581	2.4	0.06	-2.4 (-2.7, -2.0)	2.1	0.00	85.2	0.00	-85.4 (-90.7, -80.2)
Screening ECG[b]	15,642	3,241	2.1	0.34	-1.9 (-2.2, -1.6)	1.5	0.19	72.2	54.6	-14.5 (-45.2, 16.2)
Screening BMP	11,187	2,243	31.1	25.0	-5.7 (-7.7, -3.7)	28.8	18.9	92.4	75.8	-16.7 (-20.3, -13.0)
Screening LFT	17,115	3,572	12.4	10.7	-1.6 (-2.8, -0.49)	11.4	8.5	91.6	79.6	-12.0 (-16.2, -7.8)
Screening CMP	10,853	2,175	26.0	20.5	-6.0 (-7.9, -4.1)	24.1	15.4	92.7	75.1	-17.5 (-21.6, -13.4)
Screening CBC with or without differential	14,619	3,047	42.8	35.4	-7.7 (-9.6, -5.9)	40.1	27.1	93.5	76.5	-16.9 (-19.5, -14.3)
Prostate cancer screening	456	196	15.9	15.3	-0.3 (-6.3, 5.7)	15.2	12.2	95.7	80.0	-13.0 (-27.6, 1.5)
Thyroid screening	13,834	2,813	26.3	20.9	-5.5 (-7.1, -3.8)	24.6	16.0	93.7	76.7	-16.9 (-20.4, -13.4)
Screening urinalysis	17,623	3,677	5.4	4.1	-1.4 (-2.1, -0.69)	5.0	3.3	92.9	79.0	-14.2 (-20.9, -7.6)

© 2026 Ganguli I et al. *JAMA Network Open*

CRC screening	7,930	1,578	12.1	9.9	-2.1 (-3.8, -0.48)	5.3	4.7	44.0	47.1	3.8 (-4.8, 12.4)

Models used to obtain percentage point differences were adjusted for three variables for which standardized mean differences after matching were >0.1: presence of a virtual annual visit in the prior 24 months, patient insurance type, and visit quarter. Abbreviations: BMP, basic metabolic panel; CBC, complete blood count; CMP, comprehensive metabolic panel; CRC colorectal cancer; ECG, electrocardiogram; HbA_{1c}, hemoglobin A_{1c}; LFT, liver function test.

[a] High-value and low-value composites did not include exam room tests (cervical cancer screening and screening electrocardiogram). [b] 1 each of high-value and low-value cervical cancer screenings and 11 low-value electrocardiograms were ordered in virtual visits.

pp = Percentage Point; CI = Confidence Interval.

© 2026 Ganguli I et al. *JAMA Network Open*

eTable 4. Adjusted Hazard Ratio for Time to Test Completion

Test	Adjusted hazard ratio, virtual vs in-person (95% CI)
**Composite Test Categories**	
High-value[a]	0.51 (0.49, 0.52)
Low-value[a]	0.37 (0.35, 0.38)
Point-of-care laboratory	0.34 (0.33, 0.35)
Scheduled	0.86 (0.79, 0.94)
**High-Value Tests**	
Cervical cancer screening[b]	1.3 (1.1, 1.5)
HbA_{1c}	0.35 (0.33, 0.37)
Lipid panel	0.37 (0.35, 0.40)
CRC screening	0.95 (0.79,1.14)
Mammography	0.70 (0.63, 0.79)
**Low-Value Tests**	
Cervical cancer screening[b]	0.00 (0.00,0.00)
Screening ECG[b]	0.52 (0.27, 1.0)
Screening BMP	0.33 (0.31, 0.35)
Screening LFT	0.33 (0.31, 0.35)
Screening CMP	0.32 (0.29, 0.35)
Screening CBC with or without differential	0.33 (0.31, 0.35)
Prostate cancer screening	0.42 (0.25, 0.68)
Thyroid screening	0.33 (0.30, 0.36)
Screening urinalysis	0.38 (0.32, 0.46)
CRC screening	1.1 (0.84, 1.4)

Hazard ratios represent the relative likelihood, compared to a test ordered in an in-person visit, that a test ordered at a virtual visit will be completed at any given timepoint following an order. Abbreviations: BMP, basic metabolic panel; CBC, complete blood count; CMP, comprehensive metabolic panel; CRC colorectal cancer; ECG, electrocardiogram; HbA_{1c}, hemoglobin A_{1c}; LFT, liver function test.

[a] High-value and low-value composites did not include exam room tests (cervical cancer screening and screening electrocardiogram).

[b] 1 each of high-value and low-value cervical cancer screenings and 11 low-value electrocardiograms were ordered in virtual visits.

CI = Confidence Interval.

© 2026 Ganguli I et al. *JAMA Network Open*
