# Supplementary material for: Test Ordering and Completion During Virtual vs In-Person Annual Visits
Source: JAMA Netw Open. 2026 Feb 25;9(2):e260013. doi: 10.1001/jamanetworkopen.2026.0013 (PMC12936881; doi:10.1001/jamanetworkopen.2026.0013)
Supplement: Supplement 2. — Data Sharing Statement [file jamanetwopen-e260013-s002.pdf]

Data Sharing Statement

Ganguli. Test Ordering and Completion During Virtual vs In-Person Annual Visits. *JAMA Netw Open*. Published February 25, 2026. doi:10.1001/jamanetworkopen.2026.0013

Data
Data available: No

Additional Information
Explanation for why data not available: We are unable to share confidential patient data, as per our IRB agreement.
